# SingleRepExplorer: a unified interactive platform for integrated single-cell transcriptomic and immune repertoire analysis

**DOI:** 10.3389/fimmu.2026.1891118

**Published:** 2026-10-28

**Authors:** Masakazu Ishikawa

**Affiliations:** Bioinformatics Analysis Center, Kagawa University, Miki-cho, Japan

**Keywords:** BCR, cell-cell communication, clonotype, immune repertoire, multimodal integration, Shiny, single-cell RNA sequencing, TCR

## Abstract

Single-cell RNA sequencing coupled with V(D)J repertoire profiling enables simultaneous capture of lymphocyte transcriptomes and paired antigen receptor sequences, yet analysis of these multimodal datasets remains a substantial barrier for experimental immunologists. Existing graphical platforms either focus exclusively on transcriptomics or treat repertoire data as a secondary annotation, lacking the deep integration required for systems immunology. Here, I present SingleRepExplorer, a comprehensive, code-free analytical platform designed for 10x Genomics Cell Ranger output, built with R/Shiny, and distributed as a Docker container. The platform unifies transcriptomic preprocessing with Seurat, immune repertoire profiling with scRepertoire and immunarch, receptor–transcriptome multimodal correlation with CoNGA, cell-cell communication inference with CellChat, B-cell phylogenetic lineage reconstruction, CDR3 sequence-similarity networking, and CDR3 physicochemical characterization within a single interactive interface. Interface features include a Memory-Preserved Quality Control module for iterative threshold adjustment without data reload, JavaScript-based Lasso cell selection for interactive subclustering, and direct export of publication-ready editable vector graphics. I applied SingleRepExplorer to a publicly available pooled PBMC dataset from COVID-19 healthcare workers and vaccinated individuals. Without writing code, I observed a massively expanded cytotoxic T lymphocyte clonotype in the HCW_convalescent condition, characterized its diversity metrics, linked it to a transcriptionally distinct effector cluster expressing GNLY, GZMH, NKG7, and CD8A, and mapped its exploratory intercellular signaling footprint, highlighting Cluster 7-derived MIF and CCL5–CCR4 axes within the HCW_convalescent network. These illustrative results demonstrate that SingleRepExplorer enables an experimental immunologist to carry a dataset from raw sequencing output to systems-level hypotheses entirely through a graphical interface, and benchmarking of the platform’s core modules against independent reference workflows is reported alongside. The platform is freely available at https://github.com/amufaamo/SingleRepExplorer.

## 1 Introduction

The adaptive immune system coordinates protective responses through a vast repertoire of antigen receptors generated by somatic V(D)J recombination, producing virtually limitless structural diversity centered on the complementarity-determining region 3 (CDR3) (1, 2). High-throughput single-cell RNA sequencing (scRNA-seq) platforms now enable simultaneous profiling of full-length V(D)J sequences and genome-wide transcriptomes from individual cells (3, 4). This paired multimodal readout is transforming our understanding of clonal dynamics in infection (5), vaccination (6), autoimmunity (7), and cancer (8) by revealing not only which clones expand, but also the precise phenotypic states they occupy.

The 10x Genomics Chromium 5’ assay has emerged as the dominant platform for generating these paired datasets due to its high-throughput capture of both transcriptomes and V(D)J repertoires. However, extracting biological insight from these dual modalities remains technically demanding. Transcriptomic data require continuous, high-dimensional matrix operations, whereas immune repertoire data involve discrete, sequence-based string operations and categorical clonotype tracking. Bridging these distinct data structures requires proficiency in R or Python and familiarity with multiple specialized packages, including Seurat (9) for robust single-cell transcriptomics, scRepertoire (10) and immunarch (11) for comprehensive repertoire and diversity profiling, and CellChat (12) for inferring intercellular signaling. These packages are established community standards, but integrating their capabilities poses a substantial barrier for experimental immunologists.

Existing graphical platforms for scRNA-seq — including Asc-Seurat (13), Cerebro (14), ASAP (15), PIVOT (16), ShinyCell (17), CZ CELLxGENE (18), WASP (19), SCALA (20) and SEQUIN (21) — provide accessible transcriptomic interfaces, and several reach well beyond visualisation; SCALA, for instance, adds ligand–receptor analysis and regulatory network reconstruction. None of them, however, natively ingests paired V(D)J repertoire data (**Table 1**). Conversely, repertoire-focused tools such as VDJtools (22), the Change-O/Immcantation framework (23), immunarch (11) and scirpy (24) analyse receptors in depth — Immcantation additionally reconstructs B-cell clonal lineages — but they are script-driven and offer no integrated transcriptomic workbench.

**Table 1:** Feature comparison of SingleRepExplorer with existing single-cell analytical platforms.

Tool	Transcriptomics (GEX)	Repertoire V(D)J	Receptor–transcriptome coupling	Cell–cell communication	BCR clonal lineage	Code-free GUI	Editable vector export to PowerPoint
Asc-Seurat (13)	✓	–	–	–	–	✓	–
Cerebro (14)	✓	–	–	–	–	✓	–
ASAP (15)	✓	–	–	–	–	✓	–
PIVOT (16)	✓	–	–	–	–	✓	–
ShinyCell (17)	✓	–	–	–	–	✓	–
CZ CELLxGENE (18)	✓	–	–	–	–	✓	–
WASP (19)	✓	–	–	–	–	✓	–
SCALA (20)	✓	–	–	✓	–	✓	–
SEQUIN (21)	✓	–	–	–	–	✓	–
VDJtools (22)	–	✓	–	–	–	–	–
Immcantation/Change-O (23)	–	✓	–	–	✓	–	–
immunarch (11)	–	✓	–	–	–	–	–
scirpy (24)	–	✓	–	–	–	–	–
10x Loupe Browser (25)	✓	✓	–	–	–	✓	–
10x Loupe V(D)J Browser (25)	–	✓	–	–	–	✓	–
SingleRepExplorer	**✓**	**✓**	**✓**	**✓**	**✓**	**✓**	**✓**

✓ = supported; – = not supported. Capabilities were assessed from each tool’s primary publication, official documentation or user manual (consulted August 2026).

Three points of this comparison deserve to be stated explicitly rather than left to the reader. First, no analytical algorithm in SingleRepExplorer is itself new: clustering, repertoire statistics, communication inference and tree building are performed by Seurat, scRepertoire, immunarch, CellChat and msa/ape respectively, and the platform’s contribution at that level is correct parameter passing, which the benchmarking in section 3.1 tests directly. Second, what is distinctive is the combination in the final row — receptor–transcriptome coupling, cell–cell communication and B-cell lineage reconstruction reachable from the same session as clustering and clonotype analysis, without scripting — which no surveyed tool provides, together with the integration layer that keeps a single object synchronised so that a clone identified in one module is immediately addressable in every other. Third, several interface features of SingleRepExplorer are convenient but are not unique and are not claimed as such: interactive lasso selection is available in CZ CELLxGENE, SEQUIN and Loupe Browser; subsetting followed by re-clustering within the session, and re-filtering on QC thresholds without re-importing, are both available in Loupe Browser through its Reanalyze workflow.

The closest existing solution is the commercial 10x Genomics Loupe Browser (25), which does combine gene expression with imported V(D)J clonotypes in a code-free desktop interface, and which supports interactive lasso selection, filtering and re-clustering of a selected subset. It is, however, closed-source, restricted to 10x.cloupe files, and provides neither receptor–transcriptome coupling analysis, cell–cell communication inference, nor B-cell lineage reconstruction. What is therefore still unavailable is a single open, code-free environment in which repertoire data are treated as a first-class analytical dimension alongside the transcriptome, and in which the multimodal analyses that follow from that pairing are available without scripting.

Here, I present SingleRepExplorer, a platform designed to close this gap by treating transcriptomic and repertoire data as equally important analytical dimensions within a single interface. Beyond standard transcriptomic and repertoire profiling, the platform integrates five major capabilities: (i) CoNGA-based receptor–transcriptome multimodal correlation (26); (ii) CellChat-mediated ligand-receptor communication mapping (12); (iii) B-cell phylogenetic lineage reconstruction via multiple sequence alignment and neighbor-joining methods; (iv) CDR3 sequence-similarity networking using edit-distance metrics; and (v) CDR3 physicochemical annotation.

I demonstrate the complete analytical workflow using a publicly available PBMC dataset encompassing samples from COVID-19 healthcare workers (HCW) and SARS-CoV-2-naive vaccinated volunteers (27). In this dataset, samples from 3 convalescent individuals and 4 vaccinated individuals were longitudinally collected and physically pooled by condition prior to sequencing, allowing me to showcase comparative condition-level analyses (**Table 2**). Through this illustrative dataset, I walk the full analytical path—from raw data upload to the generation of testable hypotheses about clonal dynamics and intercellular signalling—entirely through the graphical interface.

**Table 2:** Structure of the demonstration dataset.

Condition label	Arm	Timepoint/meaning	Donors (n)	Cells (n)
HCW_baseline	Convalescent (HCW)	Pre-infection baseline, longitudinal	3	14,466
HCW_convalescent	Convalescent (HCW)	~30 days post-exposure, longitudinal	3	10,447
Vaccine_pre	Vaccine	Pre-vaccination baseline, SARS-CoV-2-naive	4	11,643
Vaccine_post	Vaccine	2 weeks post prime-boost	4	13,587

Cells from the individuals within each arm were physically pooled prior to droplet generation in the original study (27) and computationally demultiplexed by cell hashing; the integrated dataset re-used here retains condition-level labels only and does not resolve individual donors. Condition-level differences shown throughout are therefore a functional demonstration and cannot be generalised across donors.

## 2 Methods

### 2.1 Architecture and input formats

SingleRepExplorer is implemented in R (v4.3.1) using the Shiny framework (v1.7.4) with a bslib-based responsive UI layout. The application uses a strictly modular architecture: each analytical module is implemented as a self-contained Shiny module, stored in the module/directory, and dynamically loaded. SingleRepExplorer is specifically designed to accept 10x Genomics Cell Ranger output. The platform accepts two primary input formats: (i) a Cell Ranger-generated HDF5 file (filtered_feature_bc_matrix.h5) for *de novo* preprocessing, or (ii) a SingleRepExplorer session file (.qs2) which restores the full analysis state without re-running the preprocessing pipeline. Optionally, filtered contig annotation CSV files for TCR and/or BCR chains (e.g., annotated via IgBLAST (28)) can be uploaded alongside the HDF5 input. A built-in demonstration dataset is accessible without file upload.

### 2.2 Data input and quality control

All condition-level samples are loaded simultaneously into memory, and downstream analyses are computed per-sample and compared within a single session. A Memory-Preserved Quality Control (QC) module enables dynamic, iterative threshold adjustment without reloading data. Upon file upload, interactive QC scatter plots and violin plots show mitochondrial read percentage, gene count (nFeature_RNA), and total read count (nCount_RNA). The raw Seurat object is retained in memory, allowing users to re-run the pipeline with different thresholds without re-uploading the data.

### 2.3 Analytical workflow overview

SingleRepExplorer is organized as a four-track analytical workflow (**Figure 1**). The outputs of the Data Input module feed into three parallel analytical tracks: the Transcriptomics track provides interactive Uniform Manifold Approximation and Projection (UMAP) visualization, gene expression analysis, differential gene expression, and Slingshot-based (29) pseudotime trajectory inference; the Immune Repertoire track provides clonotype analysis, diversity quantification, alluvial clonal tracking, CDR3 sequence-similarity networking, CDR3 physicochemical characterization, and B-cell lineage reconstruction; and the Advanced Multimodal Analysis track provides CoNGA receptor–transcriptome correlation and CellChat cell-cell communication mapping.

**Figure 1:**
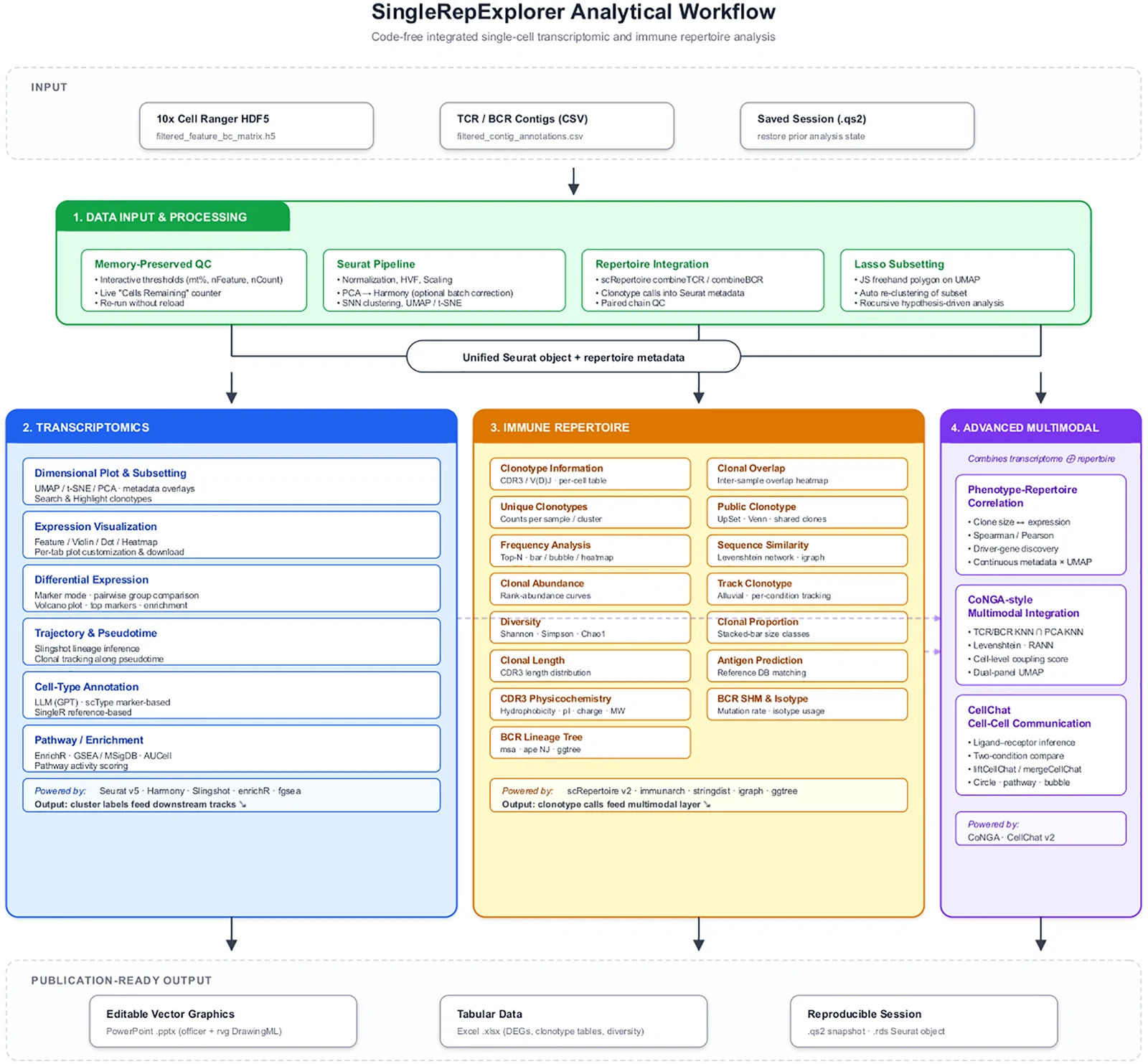
SingleRepExplorer analytical workflow. The platform integrates data input & processing (green), transcriptomics analysis (blue), immune repertoire analysis (yellow), and advanced multimodal analysis (purple) in a unified interface.

The platform depends on a curated ecosystem of R packages (Seurat v5, harmony, scRepertoire v2.0, immunarch, CellChat, msa, ape, ggtree, stringdist, igraph) bundled within a Docker image (amufaamo/singlerepexplorer:latest).

### 2.4 Transcriptomic preprocessing pipeline

The automated pipeline performs: (i) QC filtering according to user-specified thresholds; (ii) library-size normalization and log-transformation (via Seurat NormalizeData using LogNormalize with a default scale factor of 10,000, which is user-adjustable in the GUI); (iii) identification of highly variable features (default: 2,000; user-adjustable); (iv) z-score scaling; (v) principal component analysis (PCA; default: 30 dimensions; user-adjustable); (vi) optional Harmony batch correction (30); (vii) shared-nearest-neighbor (SNN) graph construction (default k = 20, user-adjustable) and community detection (default resolution: 0.5, user-adjustable); and (viii) UMAP and t-SNE dimensionality reduction. TCR and BCR contig data are integrated into the Seurat metadata upon completion.

### 2.5 Interactive dimensionality reduction and lasso subsetting

The Dimensionality Reduction module renders UMAP or t-SNE plots. A key feature is JavaScript-based Lasso selection: users draw a freehand polygon directly on the UMAP to select an arbitrary cell population, assign a user-defined label, and isolate the subset. The subset can then be automatically re-clustered within the same session.

### 2.6 Gene expression and differential expression

The Gene Expression module supports Feature Plots, Violin Plots, Dot Plots, and Heatmaps. The Differential Gene Expression (DEG) module supports one-vs-all and pairwise comparisons. Results include interactive DataTables and volcano plots with adjustable significance thresholds.

### 2.7 Pseudotime trajectory inference

Continuous developmental trajectories are inferred with Slingshot (29) applied to the user-selected dimensionality-reduction embedding (UMAP or PCA) together with the Seurat cluster labels. Users may optionally specify a starting cluster; Slingshot then fits smooth principal curves through the cluster centroids to reconstruct branching lineages and assign a per-cell pseudotime, which can be overlaid on the embedding (colored by cluster, pseudotime, clonal expansion, or a chosen gene) and exported as a table.

### 2.8 Repertoire analysis

Standard repertoire analyses are implemented using scRepertoire (10) and immunarch (11). Diversity metrics, including Shannon entropy, Simpson index, Inverse Simpson, and Chao1 richness, are computed per sample. Alluvial tracking visualizes the distribution of specific clonotypes across clusters or samples. The “Phenotype × Repertoire” scatter module correlates continuous metadata with transcriptomic embedding coordinates.

### 2.9 CoNGA multimodal integration

The CoNGA-inspired multimodal integration module (26) identifies cells simultaneously similar in TCR/BCR sequence space and transcriptomic space. The TCR/BCR sequence graph is built using Levenshtein distance between CDR3 sequences (via stringdist), and the transcriptomic graph is built using PCA embeddings (via RANN). The CoNGA score is defined as the number of shared neighbors across both graphs (user-adjustable K).

### 2.10 Cell-cell communication via CellChat

The CellChat module (12) infers intercellular ligand-receptor networks, stratified by user-selected groupings, in either a single-condition or a two-condition comparison mode. For each condition, CellChat constructs signaling probability matrices (computeCommunProb with type = “triMean”, raw.use = TRUE, nboot = 5, population.size = FALSE); in comparison mode the two objects are lifted to a common cluster space and merged before ranking pathways by relative information flow. Results are visualized as chord diagrams, heatmaps, and ligand-receptor bubble plots. The ligand–receptor database is user-selectable (Secreted Signaling, the default,/Cell-Cell Contact/ECM-Receptor/All), as is the minimum-cell threshold for cluster participation, applied via filterCommunication (default: the data-adaptive rule min.cells = ⌊N/1000⌋). To bound memory use, the module downsamples any condition exceeding a user-set cell cap (default 3,000 cells) before inference. Because my benchmarking showed this downsampling to substantially destabilise the inferred pathway ranking, the cap was raised above the population size for all analyses reported here, so that every condition was analysed in full.

### 2.11 B-cell phylogenetic lineage analysis

The BCR Lineage Tree module reconstructs intra-clonal evolutionary relationships. For a selected BCR clone, the module extracts heavy-chain nucleotide sequences, performs multiple sequence alignment with msa (31) using ClustalOmega (falling back to Muscle), computes pairwise distances between the aligned sequences as the number of differing nucleotide positions (ape::dist.dna, model “N”, pairwise deletion) (32), and builds a neighbor-joining tree (33) rooted at its midpoint, with branch lengths proportional to mutational distance. Trees are drawn with ggtree (34). Tip labels are taken from the alignment itself rather than assigned by input position, because the aligner returns sequences in guide-tree order.

### 2.12 CDR3 sequence similarity networking

The Sequence Similarity Network module computes pairwise edit distances (Levenshtein or Hamming, user-adjustable) among the top-N most abundant clonotypes’ CDR3 amino acid sequences using stringdist (35). Clonotypes within a user-defined distance threshold are connected in an undirected graph [igraph (36)] and visualized [ggnetwork (37)].

### 2.13 CDR3 physicochemical characterization

Per-CDR3 peptide properties—mean hydrophobicity (Kyte-Doolittle scale) and net charge at pH 7.0—are computed to reveal potential antigen recognition motifs. Properties are visualized as violin or box plots.

### 2.14 Publication-ready export

All modules provide download buttons that generate publication-ready editable vector graphics in PowerPoint format via officer and rvg (38), as well as raster formats (PNG, PDF) and, pending an upcoming application update, additional vector formats (SVG, TIFF). Color palettes are synchronized with Seurat’s default cluster scheme. Plot parameters are fully user-controllable.

### 2.15 Benchmarking and validation

To assess whether SingleRepExplorer’s modules are accurate and robust rather than merely executable, I benchmarked five components against reference workflows implemented independently of the application code. For most analyses the platform passes user-selected parameters directly to established community R packages (Seurat, scRepertoire, immunarch, CellChat, msa, ape). The question these benchmarks address is therefore not whether those algorithms are correct, which is established upstream, but whether the surrounding data ingest, subsetting, parameter-passing and labelling layers preserve their results — the parts of a graphical platform that can fail silently.

1. Clustering concordance: the GUI pipeline versus a manually executed reference Seurat command-line workflow, scored with the Adjusted Rand Index (ARI) (39) and Normalized Mutual Information (NMI).
2. Clonotype calling: the per-cell clonotype assignments stored by the application versus an independent scRepertoire run from the same raw filtered_contig_annotations.csv. Because clone identifiers are arbitrary strings, agreement was scored on the induced partition (ARI) rather than on label equality, together with clone-size rank correlation.
3. Diversity metrics: GUI-computed Shannon, Simpson, Inverse Simpson and Chao1 indices versus independent recomputation.
4. Cell–cell communication: (a) *accuracy* — the application’s CellChat routine versus a canonical CellChat pipeline on the identical cells and parameters; (b) *robustness* — because the module downsamples each condition to a fixed number of cells before inference, the analysis was repeated across eight downsampling seeds and three depths (1,500/3,000/5,000 cells), and the reproducibility of the inferred pathway set was quantified by pairwise Jaccard index and by the detection frequency of each pathway across replicates.
5. B-cell lineage reconstruction: (a) *recovery of known topologies* — intra-clonal somatic hypermutation was simulated along known random trees (10 or 20 variants, 400-nt heavy-chain sequences, three divergence levels, 20 replicates per condition) and the module’s reconstruction was scored against the true topology using the normalized Robinson–Foulds (nRF) distance (40), where 0 denotes an identical topology; (b) *concordance with an independent method* — the module’s neighbour-joining topology for the demonstration clone versus a maximum-parsimony reconstruction computed with phangorn (41).
6. CoNGA multimodal coupling: the CoNGA-inspired module applied to a CD8+ T cell subset, to verify that the co-neighbour score behaviour matched the expectation of the published CoNGA framework (26) for a lineage-restricted dataset.

This benchmarking was consequential rather than confirmatory: the lineage simulations exposed a defect in which tip labels were assigned positionally although the aligner returns sequences in guide-tree order, so that labels could be attached to the wrong sequences. The defect was corrected in release v1.1.1, and all lineage results reported here use the corrected implementation.

### 2.16 Dataset

I analyzed a publicly available PBMC single-cell dataset from Sureshchandra et al. (27), comprising samples from COVID-19 healthcare workers at four pooled conditions: HCW_baseline, HCW_convalescent, Vaccine_pre, and Vaccine_post. The dataset contains paired scRNA-seq, TCR, and BCR contig data processed by 10x Genomics Cell Ranger. The full condition, arm, timepoint, donor-count and cell-count definitions are given in **Table 2**, and the parameter settings used for every module in this demonstration are listed in **Supplementary Table S1**.

## 3 Results

### 3.1 Platform validation and benchmarking

Transcriptomic clustering concordance with an independent command-line Seurat workflow was high (ARI = 0.90, NMI = 0.93; both pipelines resolved 21 clusters). Clonotype calling was essentially exact: of the 29,254 cells to which the application assigned a clonotype, 29,252 were also assigned one by an independent scRepertoire run from the same raw contig file, the induced clone partitions agreed at ARI = 0.998 against both nucleotide- and amino-acid-level clone definitions, and the twenty largest clones were recovered in identical rank order (Spearman ρ = 1.00). Repertoire diversity metrics matched independent recomputation to within rounding error (Inverse Simpson: HCW_convalescent 48.6 vs 48.6, HCW_baseline 214.9 vs 215.4; all discrepancies < 0.5%). Multimodal coupling on the lineage-restricted CD8+ T cell subset yielded substantially stronger receptor–transcriptome coupling than the whole-PBMC analysis (mean CoNGA score 4.6 vs 1.8; 37.9% vs 15.4% of cells with ≥ 3 shared neighbours), reproducing the expected behaviour for transcriptionally homogeneous lineages.

For cell–cell communication, the application reproduced a canonical CellChat pipeline exactly when both were run on the same cells with the same parameters: the two runs returned an identical set of 20 signalling pathways (Jaccard index = 1.00) with perfectly concordant pathway strengths (Spearman ρ = 1.00). The inference is, however, strongly sensitive to the cell downsampling that the module applies by default to bound memory use. Repeating the analysis of one condition across eight downsampling seeds at the default depth of 3,000 cells, the number of recovered pathways ranged from 18 to 30 and the mean pairwise Jaccard index between replicate pathway sets was only 0.65. The two-condition comparison was less stable still: because it reports which of two conditions carries the greater relative information flow, the quantity that must reproduce is the *direction* of each pathway, and across the same eight seeds only MIF retained its direction in every replicate, with most pathways reversing in between one quarter and one half of replicates. Comparing the downsampled runs against the same analysis computed on all cells confirmed that downsampling both loses real pathways and manufactures spurious ones: TRAIL, IL-2, CD137 and IL-10 appeared in a subset of downsampled replicates but are absent when all cells are used.

This instability is a property of the inference at small cell numbers rather than of the interface, but it has a direct practical consequence, and I therefore computed every cell–cell communication result reported below on the complete dataset with downsampling disabled. More generally, these benchmarks argue that condition-level communication comparisons should be accompanied by a resampling check before any pathway ranking is interpreted.

For B-cell lineage reconstruction, simulations against known intra-clonal topologies showed that accuracy is governed by mutational load. At a high simulated somatic hypermutation load (0.15 substitutions per site summed over the tree) the module recovered the true topology of a ten-variant clone with a mean nRF distance of 0.17, with the topology recovered exactly in 25% of replicates; at intermediate load (0.05) mean nRF was 0.40, and at low load (0.02) it was 0.55, since a 400-nt sequence carrying only a handful of mutations cannot resolve internal branches. Reconstructions of twenty-variant clones behaved similarly but were correspondingly harder (nRF 0.31 to 0.74 across the same range). Consistent with this, the demonstration clone analysed below — whose variants differ by only 14.7 nucleotides on average — yielded a neighbour-joining topology that shared no internal split with an independent maximum-parsimony reconstruction (nRF = 1.00). The lineage module is accordingly presented as a capability demonstration, and its output should be interpreted as a resolved clonal genealogy only for clones carrying substantially deeper somatic hypermutation.

### 3.2 Data processing and immune cell landscape

The following case-study results illustrate platform functionality on a reused public dataset and are not intended as independent biological findings. The full pooled HCW and vaccine cohort dataset (27) was uploaded to SingleRepExplorer: the Cell Ranger HDF5 file together with paired TCR (29,254 cells) and BCR (9,345 cells) contig annotations. Following quality control (minimum 200 features, maximum 15% mitochondrial reads), the automated pipeline identified 21 distinct transcriptomic clusters across 50,143 high-quality cells from the four conditions: HCW_baseline (n = 14,466), HCW_convalescent (n = 10,447), Vaccine_pre (n = 11,643), and Vaccine_post (n = 13,587) (**Figure 2D**). UMAP visualization revealed well-separated immune populations (**Figure 2A**). Overlaying TCR-positive cells (58.4% of the total) showed they localized predominantly to T cell clusters (**Figure 2B**). Feature plots of canonical lineage and effector markers confirmed the expected immune cell-type assignments, with cytotoxic effector genes (GNLY, NKG7, GZMH) concentrated in a discrete upper-left cluster (**Figure 2C**).

**Figure 2:**
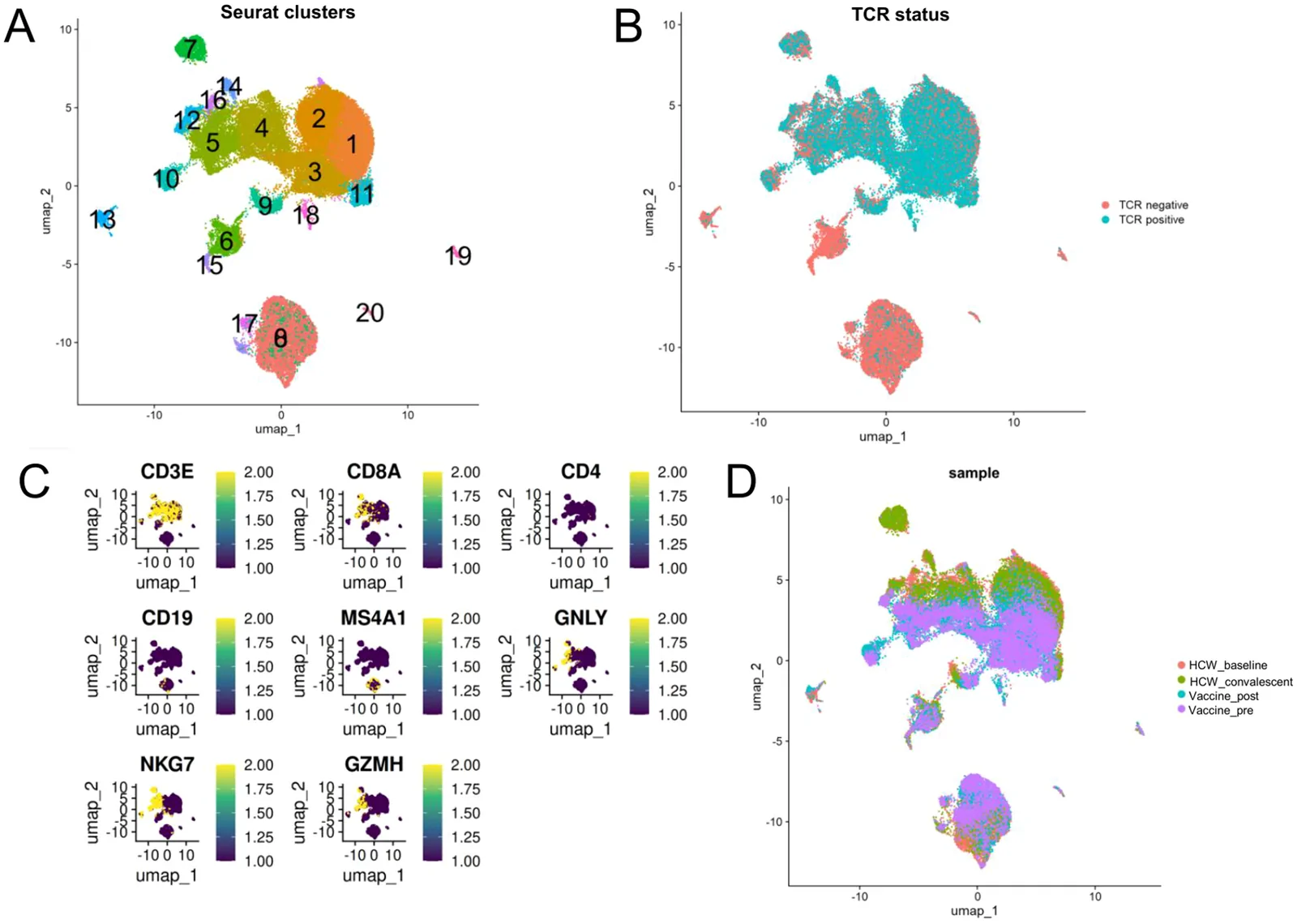
Immune cell landscape of the PBMC cohort. **(A)** UMAP colored by Seurat cluster identity (21 clusters, n = 50,143 cells). **(B)** UMAP colored by TCR status (TCR-positive in teal, TCR-negative in salmon). **(C)** Feature plots of lineage and effector markers: CD3E, CD8A, CD4, CD19, MS4A1, GNLY, NKG7, and GZMH. **(D)** UMAP colored by sample condition (HCW_baseline, HCW_convalescent, Vaccine_pre, Vaccine_post).

### 3.3 TCR repertoire analysis demonstrates detection of clonal dominance

Diversity quantification using the Inverse Simpson index revealed lower TCR diversity in the HCW_convalescent condition (Inverse Simpson = 48.6) than in HCW_baseline (215.4), Vaccine_post (235.6), and Vaccine_pre (274.7), representing an approximately 4.4-fold reduction relative to the HCW_baseline condition, a pattern compatible with clonal expansion in this condition (**Figure 3A**).

**Figure 3:**
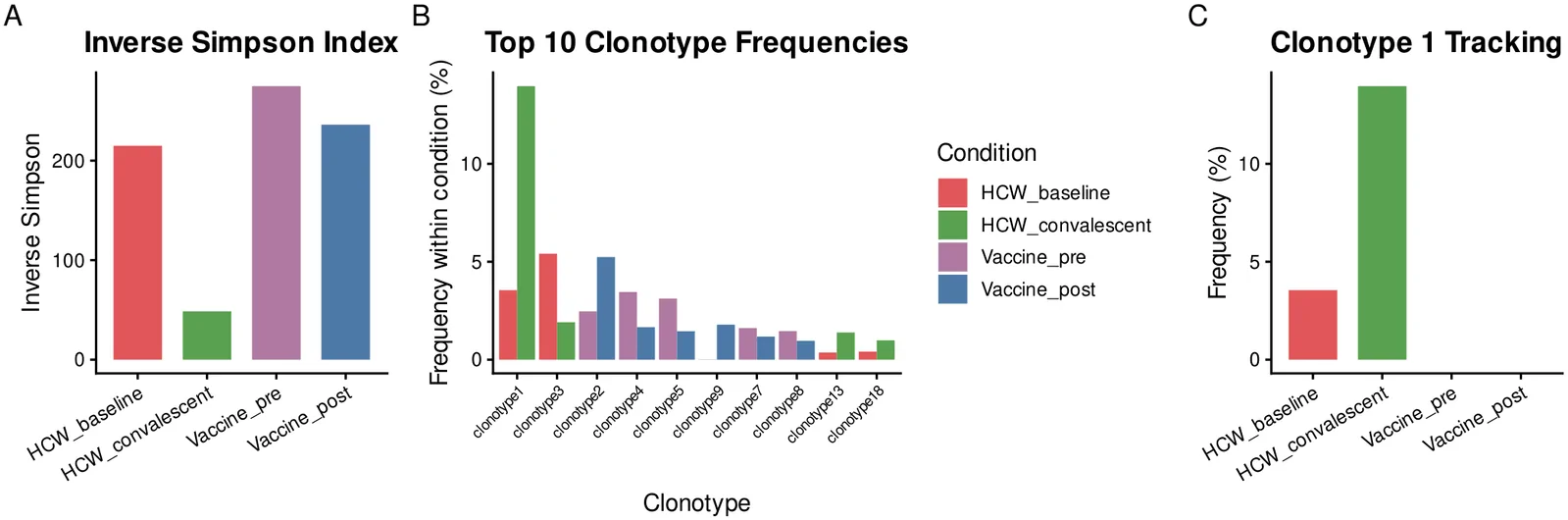
TCR repertoire analysis of clonal expansion. **(A)** Inverse Simpson diversity index across the four conditions; HCW_convalescent shows markedly reduced diversity compared to the other conditions. **(B)** Top 10 clonotype frequencies per condition (percent within each sample), showing the abundance of clonotype 1 in HCW_convalescent. **(C)** Per-condition bar tracking of clonotype 1, demonstrating enriched presence in HCW_convalescent.

Clonotype frequency analysis identified clonotype 1 (encoding paired TRBV21–1 and TRBV6–5 V-gene segments) as the most abundant clone in HCW_convalescent, reaching 14.0% of the 5,965 paired-TCR cells in that condition (833 cells). Targeted clonotype tracking confirmed that clonotype 1 was strongly enriched in HCW_convalescent, present at low frequency in HCW_baseline, and undetectable at the vaccination timepoints—a pattern of clonal expansion that the original study of this cohort attributed to SARS-CoV-2 infection (27) (**Figures 3B, C**).

### 3.4 Clonotype-transcriptome integration and DEG analysis demonstrate CTL characterization

Using the “Search & Highlight” feature, I queried clonotype 1 on the UMAP. These cells mapped almost exclusively to a single compact cluster (Cluster 7), consistent with a shared transcriptional state (**Figure 4A**). To further assess the homogeneity of this population, I used the Lasso selection tool to draw a polygon directly around the clonotype 1-enriched region on the UMAP (**Figure 4B**). The selected cells were automatically subsetted and re-clustered within the application, revealing a single homogeneous sub-cluster dominated by clonotype 1. This interactive sub-analysis demonstrates SingleRepExplorer’s capacity for recursive, hypothesis-driven investigation without coding.

**Figure 4:**
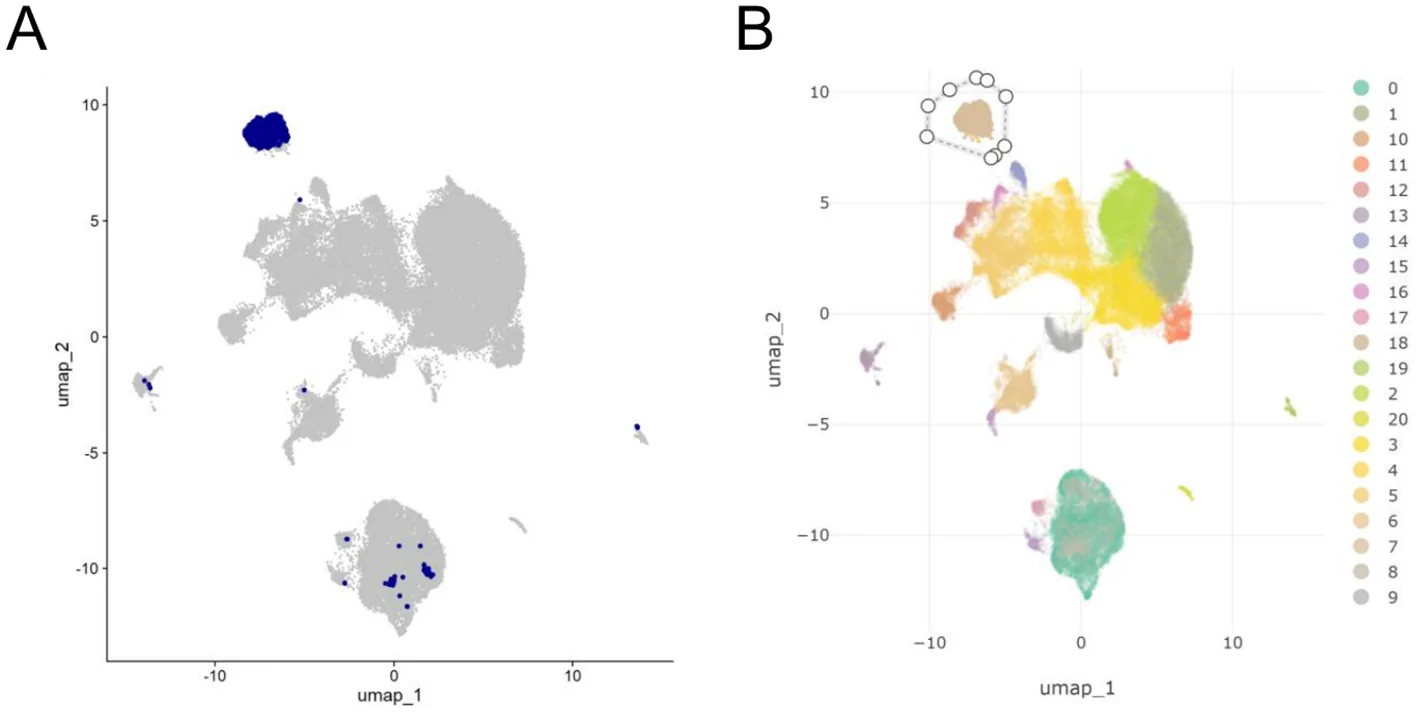
Clonotype–transcriptome integration via interactive UMAP tools. **(A)** UMAP with clonotype 1 cells highlighted in dark blue, mapping to Cluster 7. **(B)** Lasso selection interface screenshot showing interactive freehand polygon drawing directly on the UMAP for automated subsetting and re-clustering of the targeted population.

I performed DEG analysis using the “Find Cluster Markers” mode, comparing Cluster 7 with all other clusters across the full 50,143-cell dataset (**Table 3**).

**Table 3:** Top Differentially Expressed Genes (DEGs) in Cluster 7 vs. all other clusters.

Gene	avg_log2FC	pct.1	pct.2	p_val_adj	Biological role
TRBV21-1	6.56	0.949	0.021	< 1e-300	TCR V-gene (clonotype1)
TRBV6-5	5.14	0.955	0.027	< 1e-300	TCR V-gene (clonotype1)
TRGV4	4.48	0.707	0.041	< 1e-300	γδ-TCR V-gene
KIR3DL2	4.40	0.593	0.031	< 1e-300	NK receptor, terminally diff. CTL
KIR2DL3	3.85	0.575	0.040	< 1e-300	NK receptor, terminally diff. CTL
TRAV29DV5	3.65	0.767	0.055	< 1e-300	TCR α-chain V-gene
COL6A2	3.32	0.774	0.135	< 1e-300	Extracellular matrix
CMC1	2.52	0.871	0.231	< 1e-300	Cytotoxic CTL marker
KLRD1	2.34	0.788	0.175	< 1e-300	NK/CTL activation receptor
GNLY	2.26	0.889	0.211	< 1e-300	Granulysin (cytotoxic)
NKG7	2.11	0.996	0.352	< 1e-300	NK/CTL effector
GZMH	2.04	0.817	0.187	< 1e-300	Granzyme H (cytotoxic)
CD8A	1.96	0.975	0.336	< 1e-300	CD8 co-receptor
ZEB2	1.96	0.886	0.317	< 1e-300	Effector/memory transcription factor
CST7	1.83	0.981	0.382	< 1e-300	CTL degranulation
GZMB	1.79	0.630	0.146	< 1e-300	Granzyme B (cytotoxic)
KLRK1	1.78	0.809	0.283	< 1e-300	NK activating receptor NKG2D
KLRB1	1.68	0.839	0.262	< 1e-300	NKR-P1 (CD161)

Effect sizes and detection rates are as computed; all listed genes have a Benjamini–Hochberg-adjusted P value below double-precision representation and are reported as < 1e-300. The comparison used Seurat’s Wilcoxon rank-sum test on all 50,143 cells (Cluster 7, n = 1,637, versus all other clusters, n = 48,506).

The two TCR V-gene segments of clonotype 1—TRBV21–1 and TRBV6-5—ranked as the most significantly upregulated genes. KIR receptor genes (KIR3DL2 and KIR2DL3), canonically expressed by terminally differentiated NK-like CTLs, were highly elevated. Canonical cytotoxic effector molecules—GNLY, GZMH, NKG7, CD8A, and GZMB—were all strongly upregulated. The transcription factor ZEB2 (42, 43) was also consistent with an effector phenotype. At the application’s default thresholds (adjusted P < 0.05, |log2 fold change| > 0.1) the comparison returned 2,775 differentially expressed genes (1,852 up, 923 down), whose overall distribution was visualized as a volcano plot (**Figure 5A**). Dot plot visualization across all 21 clusters demonstrated that this cytotoxic gene signature was highly specific to Cluster 7 (**Figure 5B**), suggesting that clonotype 1 may represent a highly differentiated CTL population.

**Figure 5:**
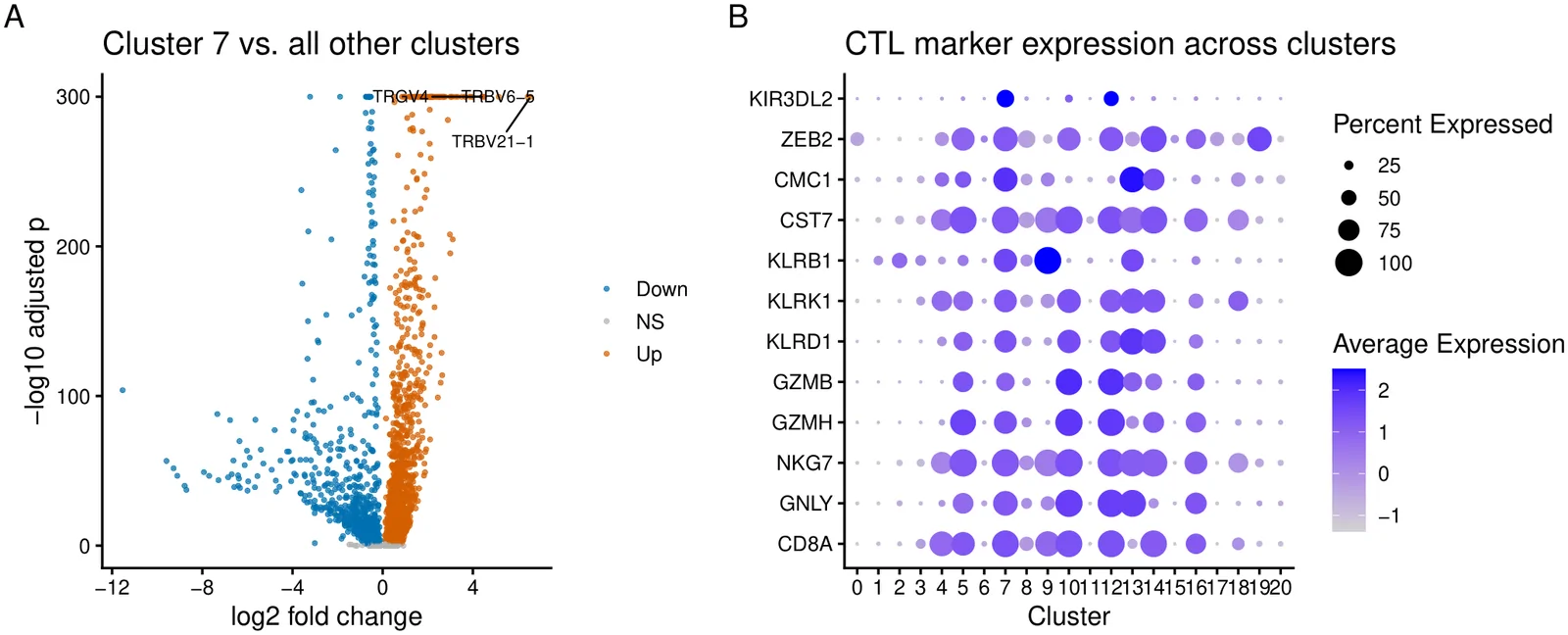
Differential gene expression analysis of the expanded CTL population, computed on all 50,143 cells. **(A)** Volcano plot of DEGs between Cluster 7 (n = 1,637) and all other clusters (n = 48,506). Cytotoxic effector genes and TCR V-gene segments are prominently upregulated. Eighty-five genes whose adjusted P value falls below double-precision representation are plotted at the axis cap of 300. **(B)** Dot plot showing expression of top CTL marker genes across all 21 clusters. Expression is highly specific to Cluster 7.

To demonstrate the platform’s capacity for phylogenetic analysis, I generated a B-cell lineage tree for a selected expanded BCR clone (clonotype 2; 11 cells; IGHV2-5/IGHJ4), whose 10 distinct heavy-chain somatic-hypermutation variants (mean pairwise edit distance 14.7 nucleotides) were resolved by neighbor-joining into an intra-clonal lineage tree (**Supplementary Figure S3**). Consistent with my functionality-demonstration scope, BCR clones in this PBMC dataset were small; the module is expected to be most informative for B-cell-focused datasets with deeper clonal families.

### 3.5 Exploratory cell-cell communication analysis

I applied the CellChat module in two-condition comparison mode to the paired HCW_baseline (14,466 cells) and HCW_convalescent (10,447 cells) conditions. These analyses were exploratory and intended to illustrate the platform’s capability for modeling complex intercellular communication. Following the benchmarking above, the module’s default cell downsampling was disabled and all cells of each condition were used. A data-adaptive minimum-cell threshold (min.cells = ⌊N/1000⌋, i.e., 14 cells for HCW_baseline and 10 for HCW_convalescent) was applied via filterCommunication, and communication probabilities were computed with nboot = 5 permutations using the Secreted Signaling database. Cluster sizes in the compared conditions spanned a wide range (e.g., the expanded CTL cluster comprised 1,205 HCW_convalescent cells).

A circle plot of TNF signaling in the HCW_convalescent condition (**Figure 6A**) illustrated the inter-cluster topology of an inflammatory pathway. Cluster 16, a small interferon-stimulated cluster expressing IFIT1, IFIT2, IFIT3, OASL and ISG15 together with TNF itself, was the dominant TNF source (outgoing strength 0.47), followed by the MAIT-like Cluster 9 (0.24); their signals converged on Clusters 19, 18 and 14. Neither is the expanded CTL cluster, illustrating that the module resolves signalling roles independently of clonal expansion.

**Figure 6:**
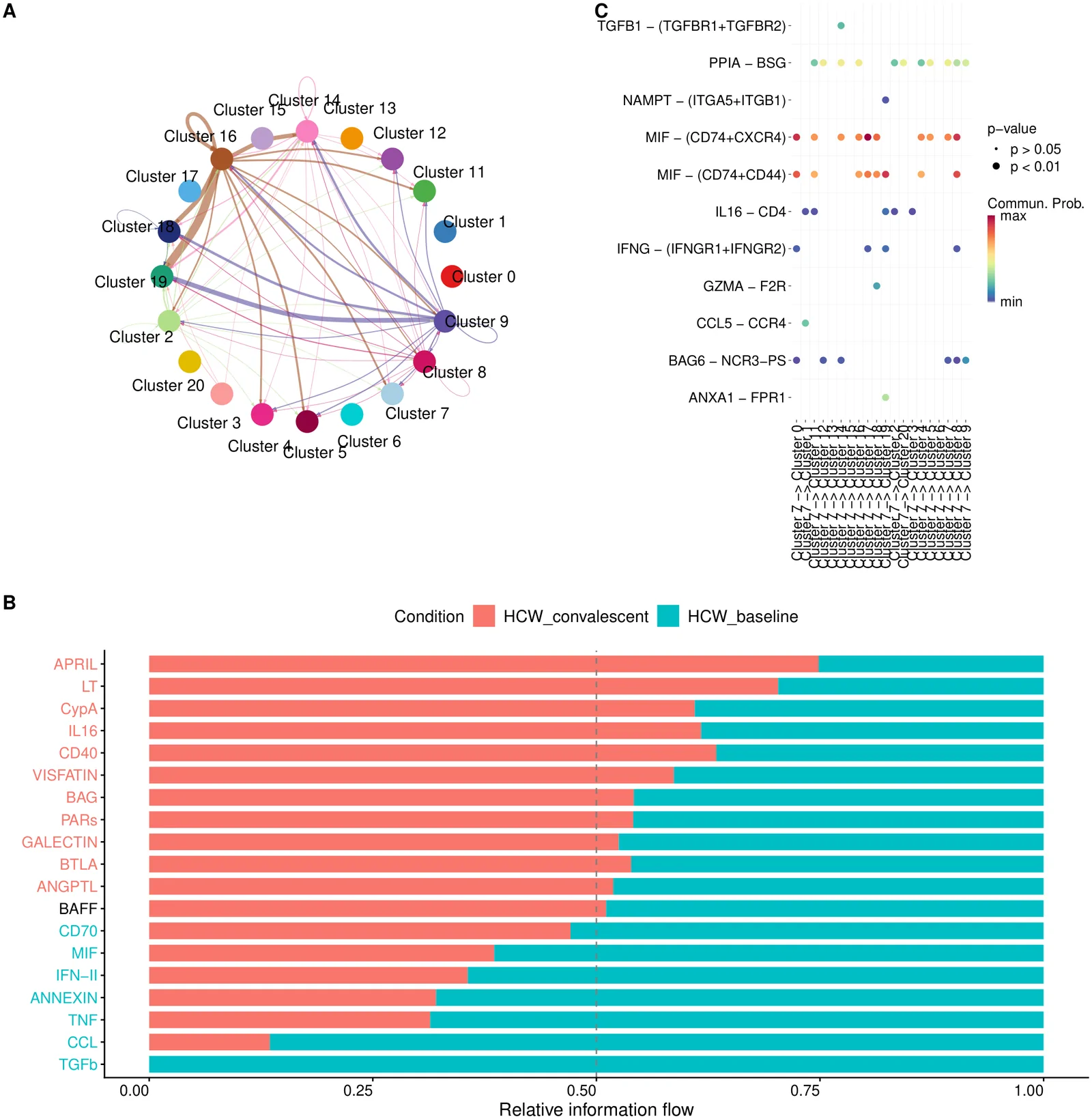
Cell–cell communication analysis, computed on all cells of each condition with the module’s default downsampling disabled. **(A)** Circle plot of TNF signaling pathway ligand–receptor interactions across Seurat clusters in HCW_convalescent. The interferon-stimulated Cluster 16 is the dominant signaling source, followed by Cluster 9. **(B)** Relative information flow per pathway between HCW_baseline and HCW_convalescent using CellChat rankNet. **(C)** Bubble plot of ligand–receptor pair probabilities originating from the expanded CTL cluster (Cluster 7) to all target clusters within HCW_convalescent. Prominent Cluster 7-derived axes include MIF–(CD74+CXCR4), MIF–(CD74+CD44), CCL5–CCR4, GZMA–F2R, and IFNG–(IFNGR1+IFNGR2).

At the pathway level (**Figure 6B**), the platform ranks signaling pathways by their relative information flow across the two conditions. Nineteen pathways were recovered. TGF-β, CCL, TNF, ANNEXIN, IFN-II and MIF carried greater relative weight in HCW_convalescent, while APRIL, LT, CD40, IL-16, CypA, VISFATIN, BAG, PARs, BTLA, GALECTIN and ANGPTL were weighted toward HCW_baseline; CD70 and BAFF were essentially balanced between conditions and are not interpreted. The largest shifts were TGF-β, which was detected only in HCW_convalescent, and CCL (relative flow 0.87 toward HCW_convalescent). As with the other case-study results, these condition-level differences illustrate the comparison workflow rather than definitive biological findings, and the pooled design precludes donor-level inference.

Within-condition ligand–receptor resolution revealed that the expanded CTL cluster (Cluster 7) was a disproportionate contributor to specific signaling axes (**Figure 6C**). Cluster 7 emitted MIF signaling via the MIF–(CD74+CXCR4) and MIF–(CD74+CD44) complexes, directed strongly toward Cluster 0, together with chemokine signaling (CCL5–CCR4), interferon-γ signaling within the IFN-II pathway, and Granzyme A signaling through the protease-activated receptor axis (GZMA–F2R). The MIF and CCL pathways were among those carrying greater relative weight in the HCW_convalescent network overall (**Figure 6B**), and this within-condition view localises a substantial share of that signal to the single expanded clonal CTL population.

### 3.6 Additional analyses demonstrating platform capability

Sequence-similarity networking and CDR3 physicochemical characterization were executed on the top clonotype panel across the four conditions (**Supplementary Figure S1**). Both modules ran successfully through the GUI, though in this illustrative dataset, they did not reveal additional biological structure beyond the transcriptomic findings reported above. The platform’s CoNGA-inspired multimodal integration module was also executed across the full dataset (**Supplementary Figure S2**). As expected for a heterogeneous PBMC mixture, scores were broadly low; I hypothesize that co-neighbor analysis is dominated by inter-lineage variance, and that the module is more informative when applied to homogeneous immune compartments.

## 4 Discussion

SingleRepExplorer addresses a critical gap in the computational immunology toolkit by providing a unified, code-free interface for integrated single-cell transcriptomic and immune repertoire analysis. The complete analytical workflow demonstrated here—spanning transcriptomic clustering, clonal diversity assessment, multimodal receptor–transcriptome correlation, cell-cell communication modeling, and CDR3 biochemical characterization—was performed entirely through the graphical interface.

A notable limitation of the case study presented here is the dataset structure. Because the original study physically pooled cells from multiple donors per condition prior to droplet generation, and the derived dataset used in my demonstration retains only condition-level labels without per-individual metadata, the analyses reflect condition-level pooled profiles rather than paired within-subject statistics. The biological differences observed between the HCW_baseline and HCW_convalescent conditions are therefore illustrative of SingleRepExplorer’s functional capabilities—demonstrating how one might track a clonal expansion (as seen in prior COVID-19 and repertoire studies (44, 45)) or map signaling networks—but are not statistically generalizable across donors.

A key distinction from existing tools is that repertoire data are treated as a first-class analytical dimension. General-purpose scRNA-seq GUI platforms provide strong transcriptome analysis but do not natively ingest V(D)J data, while repertoire toolkits require scripting; the one code-free platform that does combine both modalities, Loupe Browser, offers neither receptor–transcriptome coupling, communication inference, nor lineage reconstruction (**Table 1**). SingleRepExplorer bridges this divide, enabling hypothesis-driven investigation: identifying a clone through diversity analysis, localizing it on the UMAP, characterizing its phenotype, and contextualizing its role through CellChat mapping, all without coding.

Several interface features address practical friction points, and it is worth being precise about which are distinctive. Interactive lasso selection, subsetting followed by re-clustering, and revising QC thresholds without re-importing the data are genuinely useful, but comparable capabilities exist elsewhere — lasso selection in CZ CELLxGENE (18), SEQUIN (21) and Loupe Browser (25), and selection-driven re-clustering and re-filtering in Loupe Browser’s Reanalyze workflow — so I make no novelty claim for them. What SingleRepExplorer adds is that these operations act on an object that carries the paired repertoire annotation, so a population isolated by lasso can immediately be interrogated for clonal composition, receptor–transcriptome coupling or signalling role. Export of editable vector graphics into PowerPoint via officer and rvg (38) is, to my knowledge, not offered by the surveyed platforms, and removes a routine bottleneck in preparing figures for publication.

Robustness testing identified and resolved namespace conflicts between co-loaded R packages (e.g., %|% replaced with %||%, S4Vectors::first() explicitly qualified), illustrating the broader challenge of namespace collisions in bioinformatics deployments.

Several limitations warrant acknowledgment. First, the platform currently accepts only 10x Genomics Cell Ranger output formats. Second, I anticipate that the CoNGA-inspired module’s sensitivity is highest when applied to transcriptomically homogeneous cell subsets. Third, RNA velocity-based inference has not yet been implemented. Fourth, large datasets may approach memory limits on standard workstations. Future development will address these constraints through format adapters, modular dependency management, RNA velocity integration, and chunked processing.

In conclusion, SingleRepExplorer provides a complete, intuitive workbench for integrated single-cell immunology. It allows experimental immunologists to move from raw sequencing data to hypotheses about clonal dynamics, receptor–transcriptome coupling and intercellular signalling without writing code, and the benchmarking reported here establishes where those outputs are reliable and where — as for communication inference on small cell numbers, or lineage reconstruction on lightly mutated clones — they should be treated with caution. The platform is freely available as a Docker image and open-source R/Shiny application at https://github.com/amufaamo/SingleRepExplorer.

## Data Availability

Publicly available datasets were analyzed in this study. This data can be found here: NCBI BioProject, accession number PRJNA817521 (https://www.ncbi.nlm.nih.gov/bioproject/PRJNA817521).

## References

[1] Davis MM Bjorkman PJ . T-cell antigen receptor genes and T-cell recognition. Nature. (1988) 334:395–402. doi: 10.1038/334395a0 3043226

[2] Tonegawa S . Somatic generation of antibody diversity. Nature. (1983) 302:575–81. doi: 10.1038/302575a0 6300689

[3] Zheng GX Terry JM Belgrader P Ryvkin P Bent ZW Wilson R . Massively parallel digital transcriptional profiling of single cells. Nat Commun. (2017) 8:14049. doi: 10.1038/ncomms14049 28091601 PMC5241818

[4] 10x Genomics . Cell Ranger Software. Available online at: https://support.10xgenomics.com/single-cell-vdj/software/overview/welcome (Accessed April 27, 2026).

[5] Kuri-Cervantes L Pampena MB Meng W Rosenfeld AM Ittner CAG Weisman AR . Comprehensive mapping of immune perturbations associated with severe COVID-19. Sci Immunol. (2020) 5:eabd7114. doi: 10.1126/sciimmunol.abd7114 32669287 PMC7402634

[6] Turner JS Kim W Kalaidina E Goss CW Rauseo AM Schmitz AJ . SARS-CoV-2 infection induces long-lived bone marrow plasma cells in humans. Nature. (2021) 595:421–5. doi: 10.1038/s41586-021-03647-4 34030176

[7] Rao DA Gurish MF Marshall JL Slowikowski K Fonseka CY Liu Y . Pathologically expanded peripheral T helper cell subset drives B cells in rheumatoid arthritis. Nature. (2017) 542:110–4. doi: 10.1038/nature20810 28150777 PMC5349321

[8] Yost KE Satpathy AT Wells DK Qi Y Wang C Kageyama R . Clonal replacement of tumor-specific T cells following PD-1 blockade. Nat Med. (2019) 25:1251–9. doi: 10.1038/s41591-019-0522-3 31359002 PMC6689255

[9] Hao Y Stuart T Kowalski MH Choudhary S Hoffman P Hartman A . Dictionary learning for integrative, multimodal and scalable single-cell analysis. Nat Biotechnol. (2024) 42:293–304. doi: 10.1038/s41587-023-01767-y 37231261 PMC10928517

[10] Borcherding N Bormann NL Kraus G . scRepertoire: An R-based toolkit for single-cell immune receptor analysis. F1000Res. (2020) 9:47. doi: 10.12688/f1000research.22139.2 32789006 PMC7400693

[11] Nazarov VI Tsvetkov VO Rumynskiy E Popov AA Balashov I Volobueva M . immunarch: Bioinformatics Analysis of T-Cell and B-Cell Immune Repertoires. R package version 0.6.8 (2022). Available online at: https://immunarch.com (Accessed October 21, 2026).

[12] Jin S Guerrero-Juarez CF Zhang L Chang I Ramos R Kuan CH . Inference and analysis of cell-cell communication using CellChat. Nat Commun. (2021) 12:1088. doi: 10.1038/s41467-021-21246-9 33597522 PMC7889871

[13] Pereira WJ Almeida FM Conde D Balmant KM Triozzi PM Schmidt HW . Asc-Seurat: analytical single-cell Seurat-based web application. BMC Bioinf. (2021) 22:556. doi: 10.1186/s12859-021-04472-2 34794383 PMC8600690

[14] Hillje R Pelicci PG Luzi L . Cerebro: Interactive visualization of scRNA-seq data. Bioinformatics. (2020) 36:2311–3. doi: 10.1093/bioinformatics/btz877 31764967 PMC7141853

[15] Gardeux V David FPA Shajkofci A Schwalie PC Deplancke B . ASAP: A web-based platform for the analysis and interactive visualization of single-cell RNA-seq data. Bioinformatics. (2017) 33:3123–5. doi: 10.1093/bioinformatics/btx337 28541377 PMC5870842

[16] Zhu Q Fisher SA Dueck H Middleton S Khaladkar M Kim J . PIVOT: platform for interactive analysis and visualization of transcriptomics data. BMC Bioinf. (2018) 19:6. doi: 10.1186/s12859-017-1994-0 29304726 PMC5756333

[17] Ouyang JF Kamaraj US Cao EY Rackham OJL . ShinyCell: simple and sharable visualization of single-cell gene expression data. Bioinformatics. (2021) 37:3374–6. doi: 10.1093/bioinformatics/btab209 33774659

[18] Chan Zuckerberg Initiative . CZ Cellxgene Discover. Available online at: https://cellxgene.cziscience.com/ (Accessed August 16, 2026).

[19] Hoek A Maibach K Özmen E Vazquez-Armendariz AI Mengel JP Hain T . WASP: a versatile, web-accessible single cell RNA-Seq processing platform. BMC Genomics. (2021) 22:195. doi: 10.1186/s12864-021-07469-6 33736596 PMC7977290

[20] Tzaferis C Karatzas E Baltoumas FA Pavlopoulos GA Kollias G Konstantopoulos D . SCALA: a complete solution for multimodal analysis of single-cell Next Generation Sequencing data. Comput Struct Biotechnol J. (2023) 21:5382–93. doi: 10.1016/j.csbj.2023.10.032 38022693 PMC10651449

[21] Weber C Hirst MB Ernest B Schaub NJ Wilson KM Wang K . SEQUIN is an R/Shiny framework for rapid and reproducible analysis of RNA-seq data. Cell Rep Methods. (2023) 3:100420. doi: 10.1016/j.crmeth.2023.100420 37056373 PMC10088091

[22] Shugay M Bagaev DV Turchaninova MA Bolotin DA Britanova OV Putintseva EV . VDJtools: Unifying post-analysis of T cell receptor repertoires. PloS Comput Biol. (2015) 11:e1004503. doi: 10.1371/journal.pcbi.1004503 26606115 PMC4659587

[23] Gupta NT Vander Heiden JA Uduman M Gadala-Maria D Yaari G Kleinstein SH . Change-O: A toolkit for analyzing large-scale B cell immunoglobulin repertoire sequencing data. Bioinformatics. (2015) 31:3356–8. doi: 10.1093/bioinformatics/btv359 26069265 PMC4793929

[24] Sturm G Szabo T Fotakis G Haider M Rieder D Trajanoski Z . Scirpy: a Scanpy extension for analyzing single-cell T-cell receptor-sequencing data. Bioinformatics. (2020) 36:4817–8. doi: 10.1093/bioinformatics/btaa611 32614448 PMC7751015

[25] 10x Genomics . Loupe Browser and Loupe V(D)J Browser. Available online at: https://www.10xgenomics.com/support/software/loupe-browser (Accessed August 16, 2026).

[26] Schattgen SA Guion K Crawford JC Souquette A Barrio AM Stubbington MJT . Integrating T cell receptor sequences and transcriptional profiles by clonotype neighbor graph analysis (CoNGA). Nat Biotechnol. (2022) 40:54–63. doi: 10.1038/s41587-021-00989-2 34426704 PMC8832949

[27] Sureshchandra S Lewis SA Doratt BM Jankeel A Coimbra Ibraim I Messaoudi I . Single-cell profiling of T and B cell repertoires following SARS-CoV-2 mRNA vaccination. JCI Insight. (2021) 6:e153201. doi: 10.1172/jci.insight.153201 34935643 PMC8783687

[28] Ye J Ma N Madden TL Ostell JM . IgBLAST: An immunoglobulin variable domain sequence analysis tool. Nucleic Acids Res. (2013) 41:W34–40. doi: 10.1093/nar/gkt382 23671333 PMC3692102

[29] Street K Risso D Fletcher RB Das D Ngai J Yosef N . Slingshot: cell lineage and pseudotime inference for single-cell transcriptomics. BMC Genomics. (2018) 19:477. doi: 10.1186/s12864-018-4772-0 29914354 PMC6007078

[30] Korsunsky I Millard N Fan J Slowikowski K Zhang F Wei K . Fast, sensitive and accurate integration of single-cell data with Harmony. Nat Methods. (2019) 16:1289–96. doi: 10.1038/s41592-019-0619-0 31740819 PMC6884693

[31] Bodenhofer U Bonatesta E Horejš-Kainrath C Hochreiter S . msa: an R package for multiple sequence alignment. Bioinformatics. (2015) 31:3997–9. doi: 10.1093/bioinformatics/btv494 26315911

[32] Paradis E Schliep K . ape 5.0: an environment for modern phylogenetics and evolutionary analyses in R. Bioinformatics. (2019) 35:526–8. doi: 10.1093/bioinformatics/bty633 30016406

[33] Saitou N Nei M . The neighbor-joining method: a new method for reconstructing phylogenetic trees. Mol Biol Evol. (1987) 4:406–25. doi: 10.1093/oxfordjournals.molbev.a040454 3447015

[34] Yu G Smith DK Zhu H Guan Y Lam TT . ggtree: An R package for visualization and annotation of phylogenetic trees with their covariates and other associated data. Methods Ecol Evol. (2017) 8:28–36. doi: 10.1111/2041-210X.1262840046247

[35] van der Loo M . The stringdist package for approximate string matching. R J. (2014) 6:111–22. doi: 10.32614/RJ-2014-011

[36] Csardi G Nepusz T . The igraph software package for complex network research. InterJournal Complex Syst. (2006) 1695:1–9. Available online at: https://igraph.org (Accessed October 21, 2026).

[37] Briatte F . ggnetwork: Geometries to plot networks with ggplot2. R package (2016). Available online at: https://CRAN.R-project.org/package=ggnetwork (Accessed October 21, 2026).

[38] Gohel D . officer: manipulation of Microsoft Word and PowerPoint documents; rvg: R graphics devices for Office vector graphics output. R packages. Available online at: https://CRAN.R-project.org/package=officer (Accessed August 16, 2026).

[39] Hubert L Arabie P . Comparing partitions. J Classif. (1985) 2:193–218. doi: 10.1007/BF0190807530311153

[40] Robinson DF Foulds LR . Comparison of phylogenetic trees. Math Biosci. (1981) 53:131–47. doi: 10.1016/0025-5564(81)90043-2

[41] Schliep KP . phangorn: phylogenetic analysis in R. Bioinformatics. (2011) 27:592–3. doi: 10.1093/bioinformatics/btq706 21169378 PMC3035803

[42] Dominguez CX Amezquita RA Guan T Marshall HD Joshi NS Kleinstein SH . The transcription factors ZEB2 and T-bet cooperate to program cytotoxic T cell terminal differentiation in response to LCMV viral infection. J Exp Med. (2015) 212:2041–56. doi: 10.1084/jem.20150186 26503446 PMC4647261

[43] Omilusik KD Best JA Yu B Goossens S Weidemann A Nguyen JV . Transcriptional repressor ZEB2 promotes terminal differentiation of CD8+ effector and memory T cell populations during infection. J Exp Med. (2015) 212:2027–39. doi: 10.1084/jem.20150194 26503445 PMC4647262

[44] Jiang N He J Weinstein JA Penland L Sasaki S He XS . Lineage structure of the human antibody repertoire in response to influenza vaccination. Sci Transl Med. (2013) 5:171ra19. doi: 10.1126/scitranslmed.3004794 23390249 PMC3699344

[45] Meckiff BJ Ramírez-Suástegui C Fajardo V Chee SJ Kusnadi A Simon H . Imbalance of regulatory and cytotoxic SARS-CoV-2-reactive CD4+ T cells in COVID-19. Cell. (2020) 183:1340–1353.e16. doi: 10.1016/j.cell.2020.10.001 33096020 PMC7534589

