# Supplementary material for: SingleRepExplorer: a unified interactive platform for integrated single-cell transcriptomic and immune repertoire analysis
Source: Front Immunol. 2026 Oct 28;17:1891118. doi: 10.3389/fimmu.2026.1891118 (PMC13752213; doi:10.3389/fimmu.2026.1891118)
Supplement: Supplementary file 1 [file Table1.docx]

## 1.1 Supplementary Material

**Supplementary Table S1. Parameter settings used for every module in the demonstration analysis. Values are the application defaults unless noted; all are user-adjustable in the interface. Where a setting differs from the default it is marked, with the reason given.**

| **Module** | **Setting** | **Value** |
| --- | --- | --- |
| Quality control | Minimum features per cell | 200 |
|  | Maximum mitochondrial reads | 15% |
| Normalisation | Method / scale factor | LogNormalize / 10,000 |
| Feature selection | Highly variable features | 2,000 |
| Dimensionality reduction | Principal components | 30 |
|  | Batch correction (Harmony) | not applied |
| Clustering | SNN k / resolution | 20 / 0.5 |
|  | Clusters resolved | 21 |
| Repertoire | Clonotype assignment | scRepertoire combineTCR / combineBCR, filterMulti = TRUE, removeNA = TRUE |
|  | Diversity indices | Shannon, Simpson, Inverse Simpson, Chao1 |
| Differential expression | Test / thresholds | Wilcoxon; adjusted P < 0.05, \|log2 FC\| > 0.1 |
| CoNGA coupling | Neighbours (K) / mode | 15 / clonal |
| Cell–cell communication | Database | Secreted Signaling |
|  | Probability model | triMean, raw.use = TRUE, nboot = 5, population.size = FALSE |
|  | Minimum cells per cluster | ⌊N/1000⌋ = 14 (HCW_baseline), 10 (HCW_convalescent) |
|  | Cell cap per condition | disabled — all cells used (default 3,000; see section 3.1) |
| BCR lineage | Alignment / distance / tree | ClustalOmega / differing positions (dist.dna "N") / neighbour-joining, midpoint-rooted |
| Sequence-similarity network | Sequence / metric | CDR3 amino acid; Levenshtein or Hamming edit distance (stringdist) |
| CDR3 properties | Scales | Kyte–Doolittle hydrophobicity; net charge at pH 7.0 |

**Supplementary Figure S1.** CDR3 sequence similarity and physicochemical characterization of the top 50 TCR clonotypes. **(A)** Sequence similarity network. **(B)** Pairwise edit-distance heatmap. **(C)** CDR3β hydrophobicity distribution (Kyte-Doolittle mean score). In this PBMC dataset, sample-condition distributions were broadly comparable, illustrating that these modules execute successfully but require datasets with stronger expected contrasts.

**Supplementary Figure S2.** CoNGA-inspired multimodal integration. UMAPs colored by CoNGA score. The broadly low scores are consistent with expectations for heterogeneous PBMC mixtures, suggesting clearer receptor–transcriptome concordance would emerge in homogeneous immune compartments.

**Supplementary Figure S3.** B-cell heavy-chain lineage tree (capability demonstration). Neighbor-joining tree of the 10 distinct IGH somatic-hypermutation variants within an expanded BCR clone (clonotype 2; 11 cells; IGHV2-5/IGHJ4), built from the number of differing nucleotide positions between full-length heavy-chain sequences aligned with ClustalOmega (ape::dist.dna, model "N") (mean pairwise distance 14.7 nt; scale bar = 5 nucleotide differences). This illustrates reconstruction of intra-clonal mutational relationships directly from 10x Cell Ranger output; the small BCR clone sizes in this PBMC dataset reflect its use as a functionality demonstration rather than a B-cell-focused study.
